# Pre-processing of Sub-millimeter GE-BOLD fMRI Data for Laminar Applications

**DOI:** 10.3389/fnimg.2022.869454

**Published:** 2022-05-04

**Authors:** Patricia Pais-Roldán, Seong Dae Yun, N. Jon Shah

**Affiliations:** ^1^Institute of Neuroscience and Medicine 4, Medical Imaging Physics, Forschungszentrum Jülich, Jülich, Germany; ^2^Institute of Neuroscience and Medicine 11, Molecular Neuroscience and Neuroimaging, Jülich Aachen Research Alliance, Forschungszentrum Jülich, Jülich, Germany; ^3^Jlich Aachen Research Alliance, Brain - Translational Medicine, Aachen, Germany; ^4^Department of Neurology, Rheinisch-Westfälische Technische Hochschule Aachen University, Aachen, Germany

**Keywords:** noise, brain imaging, spatial specificity, gradient-echo functional MRI, data cleanup

## Abstract

Over the past 30 years, brain function has primarily been evaluated non-invasively using functional magnetic resonance imaging (fMRI) with gradient-echo (GE) sequences to measure blood-oxygen-level-dependent (BOLD) signals. Despite the multiple advantages of GE sequences, e.g., higher signal-to-noise ratio, faster acquisitions, etc., their relatively inferior spatial localization compromises the routine use of GE-BOLD in laminar applications. Here, in an attempt to rescue the benefits of GE sequences, we evaluated the effect of existing pre-processing methods on the spatial localization of signals obtained with EPIK, a GE sequence that affords voxel volumes of 0.25 mm^3^ with near whole-brain coverage. The methods assessed here apply to both task and resting-state fMRI data assuming the availability of reconstructed magnitude and phase images.

## Introduction

The recent increase in the availability of ultra-high-field (≥7 Tesla) magnetic resonance imaging (MRI) scanners has led to a growing collection of functional studies employing high-resolution methods to investigate brain function with unprecedented detail (Huber et al., 2017, 2020a,b; Kashyap et al., 2018b; Marquardt et al., 2018; Lawrence et al., 2019; Mishra et al., 2019; Moerel et al., 2019; Poplawsky et al., 2019; Self et al., 2019; Sharoh et al., 2019; Chai et al., 2020; Persichetti et al., 2020; Fukuda et al., 2021). In recent years, gradient-echo (GE) sequences have been regarded as the gold standard method to study brain activity with functional MRI (fMRI); and the blood-oxygen-level-dependent (BOLD) signal, highly sensitive to neuronal activation, remains the most employed contrast in the fMRI field (Ogawa et al., 1990, 1993; Turner et al., 1993). With an appropriate study design, GE-BOLD can detect neuronal responses with relatively good spatial specificity, as demonstrated in ocular dominance studies (Menon et al., 1997; Polimeni et al., 2010; Zaretskaya et al., 2020). However, GE sequences are not ideal for applications requiring accurate source localization, e.g., laminar fMRI. This owes to the complexity of the measured GE-BOLD signal (Zhang et al., 2018; Havlicek and Uludag, 2020). Neuronal activation is coupled to increased blood flow, leading to a relative decrease in deoxyhemoglobin, which compensates for the enhanced oxygen consumption in the activated area; this results in a higher MRI signal in T2/T2^*^-weighted images. The active neuronal site is surrounded by a capillary network, initially responsible for the observed vascular (BOLD) response, which spreads to downstream venules where field inhomogeneities derived from deoxygenated blood are strong. As a consequence, remote voxels that are adjacent to the macro-vasculature, especially near vessels with ‘ordered' trajectories, e.g., ascending veins (perpendicular to the cortex) or pial veins (tangential to the cortex), also exhibit signal changes related to the initial neuronal activation. The interpretation of the BOLD signal in laminar GE-fMRI applications is especially vulnerable to source mislocalisation due to the heterogeneous distribution of the venous blood across the cortical thickness. If no particular corrections are applied to the T2^*^-weighted images, laminar fMRI often renders activation profiles that are biased toward the surface of the cortex. Non-BOLD contrasts offer a less adulterate signal. For instance, the changes in cerebral-blood volume (CBV) (Belliveau et al., 1991) are specifically related to arterioles, which dilate locally in response to the firing of neighboring neuronal populations, and measurements of the cerebral-blood flow (CBF) mainly reflect the change in flow that occurs in arterioles and venules surrounding the active area (Ogawa et al., 1992; Williams et al., 1992). In contrast, upstream from the active neuronal site, the hemodynamic changes resulting from arteriole dilation and from the increased blood flow in the surrounding vessels, together with the specific cerebral metabolic rate of oxygen, all contribute to the measured BOLD signal (Ogawa et al., 1992; Shen et al., 2008).

At ultra-high field, spin-echo (SE) and GE sequences measuring BOLD-contrast are mostly sensitive to the effects from vessels in the neighboring parenchyma (extra-vascular effects) and not to the hemodynamic changes occurring directly in the venous blood (intra-vascular effect); this is due to the short T2 relaxation rate of blood at high magnetic fields. While SE sequences are sensitive to the extra-vascular effects produced by the micro-vasculature, i.e., capillaries supplying blood to local neurons (ideal scenario), GE sequences detect both micro and macro-vascular effects, which are relevant to local neuronal activation but also to downstream effects, yielding poorer signal localization. SE-based methods largely avoid large-vein contributions to the recorded BOLD signal by refocusing the effects of field inhomogeneities in the large veins using a 180° pulse (i.e., diffusion-dependent T2 contrast). A similar approach can be applied *post-hoc* to images acquired with GE-BOLD contrast (during pre-processing), which consists of regressing out the phase signal changes relevant to magnetic field distortions produced by large veins but not to the small and randomly oriented capillaries in the parenchyma (Menon, 2002; Curtis et al., 2014).

In contrast to other high-resolution methods, GE is a very efficient sequence that produces images with high signal-to-noise ratio, allowing the minimum voxel size to be reduced, fast acquisitions and the potential to cover the whole-brain with an extraordinary spatial resolution. Given its multiple advantages, the optimization of the GE pre-processing pipeline to improve signal localization will likely have a critical impact on the usability of high-resolution/sub-millimeter GE sequences in precision-sensitive applications such as laminar fMRI.

The usual fMRI pre-processing includes realignment followed by a number of filters and regression steps to correct for partial volume effects, penalize head displacement or reduce spectral features not compatible with neuronal oscillations (Caballero-Gaudes and Reynolds, 2017; Esteban et al., 2019; Drew et al., 2020); smoothing is generally avoided in high-resolution applications to minimize spatial mislocalization (Kay et al., 2019). High-resolution GE-BOLD fMRI could benefit from additional phase-based correction methods (Curtis et al., 2014). In order to assess the effect of currently available pre-processing methods on the performance of high-resolution GE-BOLD, this work applied twelve different pre-processing approaches to the fMRI signals acquired during resting-state or during the performance of a motor task in a group of healthy volunteers. The performance of each pre-processing approach was evaluated in terms of amplitude, signal heterogeneity across the cortical thickness, gray matter activation profiles, and network connectivity mapping. This work may serve as a reference to pre-process high-resolution images with existing methods that exploit the advantages of GE-sequences in the field of laminar research.

## Methods

### Subjects

Data from thirteen healthy adult volunteers (eleven males and two females; age, 29.5 ± 6 years) are included in this study. The experimental methods were approved by the local institutional review board (EK 346/17, RWTH Aachen University, Germany). All subjects underwent MR-safety screening and signed an informed written consent document before fMRI acquisition. A pneumatic belt was positioned around the subject's chest, and a pulse oximeter was placed on the 2nd, 3rd, or 4th finger of the left hand to record physiological signals during the fMRI acquisition.

### Experimental Design

Two scans were obtained from each volunteer, a resting-state fMRI (rs-fMRI) scan and a task-fMRI scan. The rs-fMRI scan lasted ~10 min; subjects were instructed to remain awake with their eyes closed and without thinking about anything in particular. The task-fMRI scan lasted ~8 min and 38 s, during which subjects performed a finger-tapping task following the protocol: (21 s index finger movement, 21 s rest) × 12. One of the volunteers was additionally scanned in a different session and performed two different tasks: “right index movement”, and “right index movement with thumb touch”, following the protocol: (21 s task, 21 s rest) × 8.

### fMRI Data Acquisition

MRI data were collected on a Siemens Magnetom Terra 7T (Siemens Healthineers, Erlangen, Germany) scanner with a circular polarized transmit head coil integrating 32 receive elements (Nova Medical, Inc., Wilmington, USA). Functional MRI data were obtained using GE EPI with keyhole (EPIK) combined with a TR-external EPI phase correction scheme; the sequence performance has been described elsewhere (Zaitsev et al., 2001, 2005; Shah and Zilles, 2003, 2004; Yun et al., 2013, 2019, 2020; Yun and Shah, 2017, 2020; Caldeira et al., 2019; Shah et al., 2019); briefly, EPIK enables a higher temporal and spatial resolution through the use of a sophisticated data sharing scheme that employs a sliding window to minimize autocorrelations in the fMRI time series data. By virtue of its higher bandwidth in the phase-encode direction, EPIK is also somewhat immune to geometrical distortions. The parameters employed for EPIK were: TR/TE = 3,500/22 ms, FA = 85°, partial Fourier = 5/8, 3-fold in-plane/3-fold inter-plane (multi-band) acceleration, matrix = 336 × 336 × 123 slices, voxel size = 0.63 × 0.63 × 0.63 mm^3^. B0 shimming was performed with a standard routine provided by the manufacturer.

### Pre-processing Pipelines

**Figure 1:**
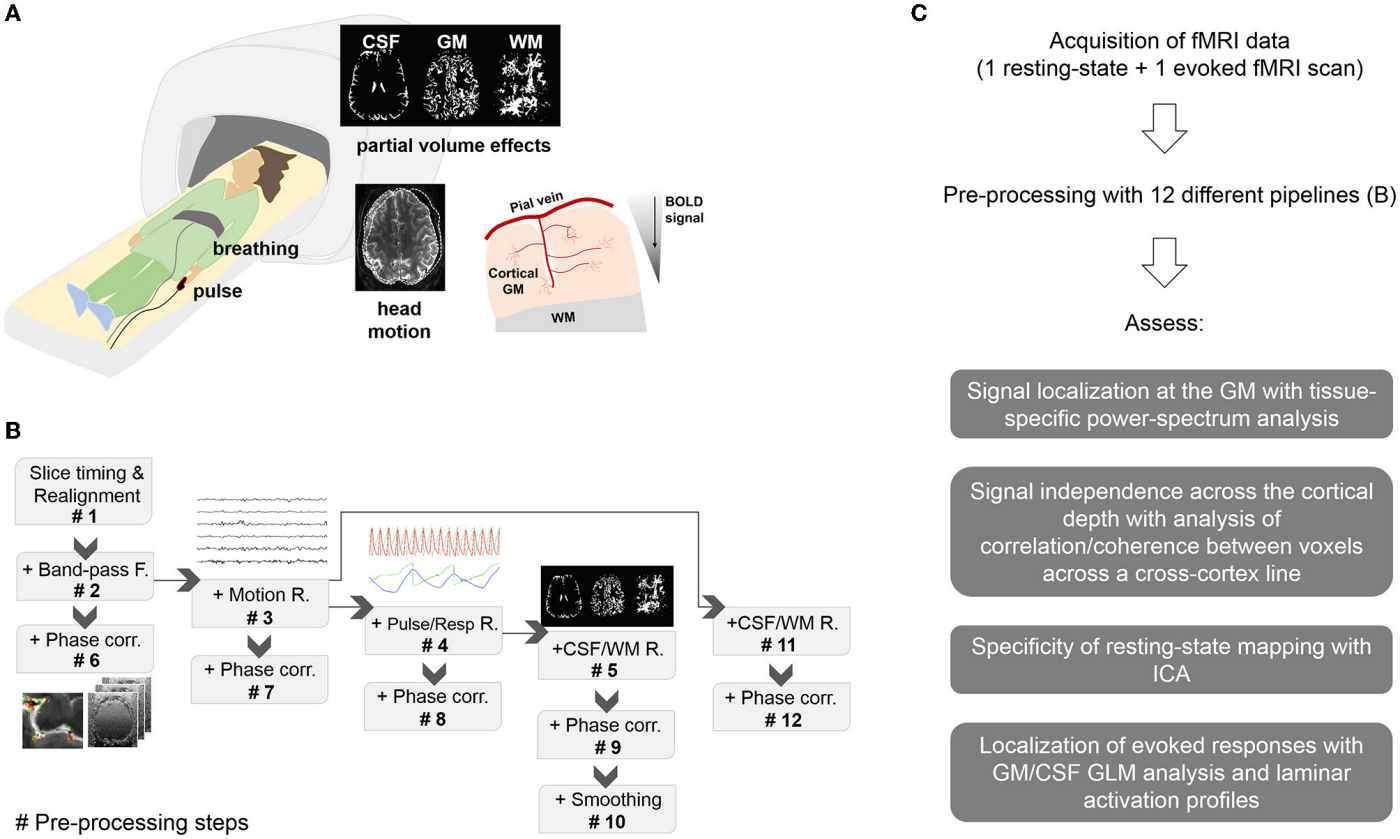
Methodology. **(A)** Experimental setup and potential sources of signal contamination. Subjects undergoing fMRI were monitored with a pulse oximeter and a pneumatic belt. Spurious signals in the high-resolution fMRI data can be derived from head movements, breathing, heart beating, partial volume effects and venous influence on the parenchymal cortical GM, typically observed as a signal decay between the cortical surface and deeper territories. **(B)** Pre-processing pipelines. Pre-proc. #*N*, with *N* = 1…5 refers to a pipeline that includes all the previous steps up to *N*. Pre-proc. #6 to #9 add phase-based vein correction to the previous pipelines. Pre-proc. #10 adds smoothing to the corrected data #9 for comparison. Pre-proc #11 and #12 add partial volume correction and phase-based correction, respectively, to Pre-proc #3. **(C)** Diagram showing the summarized workflow of the present study.

Each of the following pre-processing pipelines was applied to every functional scan from all volunteers (see Figure 1):

#1. Realignment
#2. Realignment + Bandpass (BP)-filtering
#3. Realignment + BP-filtering + Motion regression
#4. Realignment + BP-filtering + Motion regression + Physiological regression
#5. Realignment + BP-filtering + Motion regression + Physiological regression + CSF, WM regression
#6. Realignment + BP-filtering + Phase correction
#7. Realignment + BP-filtering + Motion regression + Phase correction
#8. Realignment + BP-filtering + Motion regression + Physiological regression + Phase correction
#9. Realignment + BP-filtering + Motion regression + Physiological regression + CSF,WM regression + Phase correction
#10. Realignment + BP-filtering + Motion regression + Physiological regression + Phase correction + smoothing
#11. Realignment + BP-filtering + Motion regression + CSF, WM regression
#12. Realignment + BP-filtering + Motion regression + CSF, WM regression + Phase correction

Before realignment, volumes were corrected for differences in slice acquisition times using SPM12 (Statistical Parametric Mapping Software, UCL, London, UK). A brain mask was generated from the first fMRI acquisition volume using *bet* (FSL, FMRIB Software Library, Oxford, UK). After realignment, a masked average fMRI image was obtained. This image was then subjected to tissue segmentation using *fast* (FSL), and a 99% threshold was applied to the resulting images—CSF, WM, GM maps—to create tissue-specific masks, i.e., voxels with a proportion of a certain tissue type above 99% were included in the corresponding tissue mask. The physiological signals were pre-processed using a Matlab (Matlab, Mathworks, Natick, MA, USA) implementation of RETROICOR (Glover et al., 2000); briefly, the cardiac and respiratory cycles were detected in the recorded signals, the specific cycle phase at any given TR was calculated, and a combination of sine and cosine Fourier series up to the 5th order were fit to the data to generate thirty-six physiological regressors (https://github.com/tesswallace/retroicor/blob/master/mod_retroicor.m). Additionally, the time course of the raw respiratory and cardiac traces down-sampled to 1/TR (i.e., aliased) were included in the correlation analysis for inspection. The first four acquisition volumes were removed to ensure signal stabilization. Most of the pre-processing steps were performed using AFNI (Analysis of Functional NeuroImages, NIH, Bethesda, MD) as described below.

*Realignment*. The function *3dVolreg* was used to align all volumes in the magnitude image to the first volume using six motion parameters (three rotations and three translations); then, the registration matrix was applied to the phase images using *3dAllineate*.

*Filtering*. A bandpass filter was created with *1dBport* between 0.005 and 0.12 Hz and applied to the data using *3deconvolve* with polynomials of degree 5.

*Partial volume correction*. To remove signal contamination from the CSF and WM, the time courses of voxels within the corresponding tissue mask were averaged in the realigned magnitude image, and the mean CSF and WM time courses were used as regressors of no interest.

*Regression* of the motion parameters, physiological signals and brain tissue was performed using *3dDeconvolve*, and, when applicable, several regressions were applied in a single step, to minimize blurring.

*Smoothing* consisted of applying a 1 mm full-width-half-maximum Gaussian blur to the pre-processed volumes, using *3dmerge*. One millimeter was chosen as a rather conservative blur which could still be appropriate in some high resolution applications.

*Phase-based correction*. The de-veining procedure was carried out following Menon's previous work (Menon, 2002; Curtis et al., 2014); briefly, it consisted of:

1. Applying the same pre-processing conducted in the magnitude image to the phase image (in the case of realignment, the motion parameters were calculated from the magnitude image and applied to both, magnitude and phase images). Our phase images did not show any visible wrap, i.e., no difference was found when comparing the reconstructed phase images to the result of applying a two-dimensional unwrapping function. Therefore, additional phase unwrapping was not performed.
2. Applying a linear *Fit* to the phase image, using *3dTfitter* (AFNI), so that *M* = *Fit*ϕ + *residuals*, where M = time course of the signal in the magnitude image, and = time course of the signal in the phase image. Fitϕ may be interpreted as the part of the magnitude image that can be explained by changes in the phase image, presumably related to field inhomogeneities around large vessels.
3. Subtracting Fitϕ from the magnitude image using *3dcalc*.

### Data Analysis

The different sets of data, each processed with a different pre-processing pipeline, were compared in terms of the power spectrum and signal separation in voxels along lines crossing the cerebral cortex, hereafter referred to as cross-cortex lines, which was selected on the mean EPIK image (i.e., blindly to the location of meaningful fMRI responses) as described below. Evoked data were further analyzed to compare GM vs. CSF activation and by measuring the activation profiles along a cross-cortex line.

The masks for the cross-cortex lines for analysis of rs-fMRI data were generated in three cortical regions (frontal, parietal and occipital cortex) using a combination of LayNii (Huber et al., 2021) (https://doi.org/10.5281/zenodo.3514297) and AFNI. For each selected region, a number between 4 and 20 lines were produced (depending on the size of the ROI for each participant), yielding a total of 280 lines in the 13 subjects. The cross-cortex lines contained a maximum of ten voxels, starting in the CSF, crossing the cortical GM and reaching the WM.

The relationship between the different regressors and the magnitude and phase images was assessed by computing the correlation coefficient between pairs of time courses in Matlab. The correlation matrix in Supplementary Figures 1, 2B includes, in order, the following 65 components: three head rotations, three head translations, one aliased (sampled with TR) pulse trace, one aliased respiration, ten pulse-retroicor cosine terms, ten respiration-retroicor cosine terms, sixteen multiplicative pulse-respiration terms, the average CSF, GM and WM time course, the magnitude time course from ten voxels (arranged from CSF to WM) and the phase signal from ten voxels (from CSF to WM). The correlation matrices from all subjects were averaged to obtain a mean correlation matrix. Similarly, a correlation matrix that included the time course of ten voxels along the cross-cortex lines from each pre-processing pipeline was computed to evaluate the remaining dependence of the data on each regressor. The mean correlation with noise was computed for each pipeline, i.e., averaging across regressors and across voxels, to compare the amounts of residual noise in the data. Paired *t*-tests were computed to identify statistically significant differences across pipelines.

**Figure 2:**
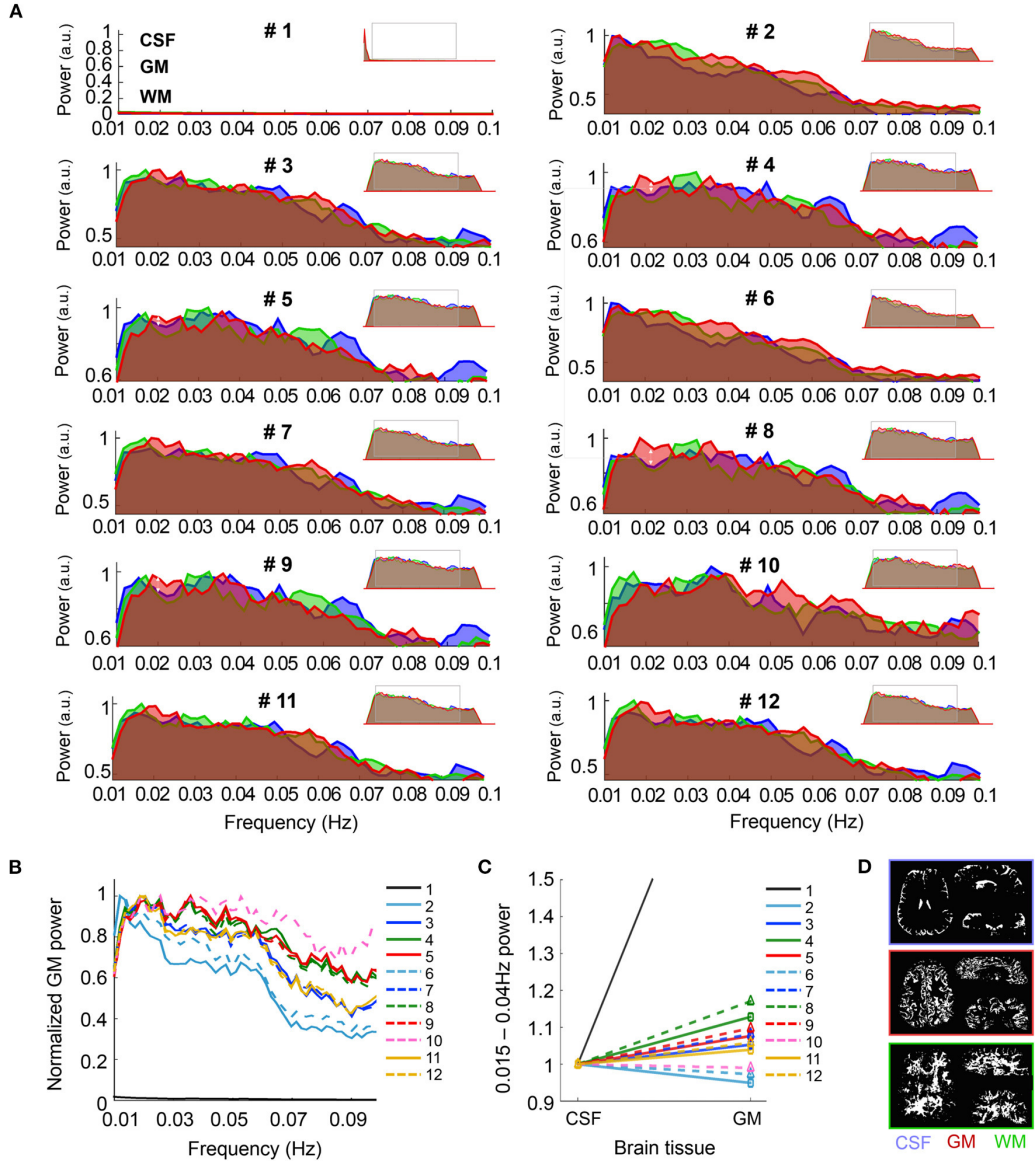
Spectral characteristics of CSF, GM and WM upon different pre-processing. **(A)** The graphs show the frequency-power decomposition of the in CSF (blue), GM (red) and WM (green) that results from applying different pre-processing steps to the data. Minimized spectra in the upper right corner of each graph represent the full spectrum (0.001–0.14 Hz). **(B)** The graph shows the normalized power of the fMRI signal in the GM after the twelve different pre-processing approaches. Note that the normalized signal in Pre-proc. #1 (black line) is near zero due to its maximum value being below 0.01 Hz. **(C)** Average power of the frequency range 0.015–0.04 Hz with respect to the whole spectrum for CSF and GM, normalized to the CSF value, upon different pre-processing. Note the higher GM/CSF ratio obtained after adding phase-regression (dashed lines vs. solid lines, for each color). **(D)** Example of the tissue-specific masks generated automatically from the mean functional image in one volunteer. *N* = 13 healthy volunteers [for **(A–C)**].

**Figure 3:**
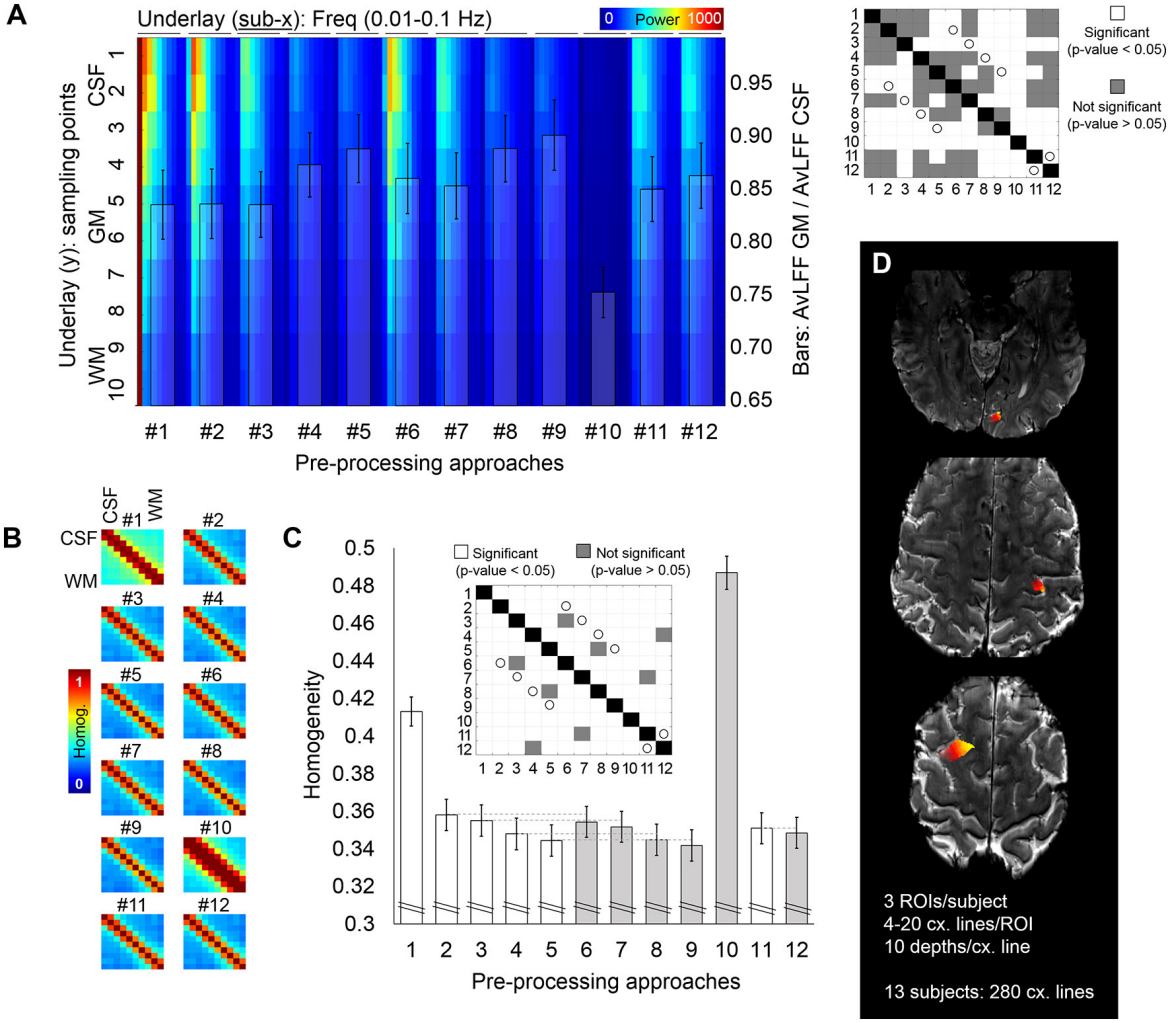
Cross-cortex evaluation of the resting-state fMRI signal. **(A)** Whole spectrum along ten voxels crossing the cortical ribbon, from CSF to WM (jet color-coded underlay) and ratio of the power of the 0.015–0.04 Hz frequency band for GM with respect to CSF (bar plot overlay) quantified from fMRI data pre-processed with twelve different pre-processing pipelines. The matrix on the right shows, in white, significant differences relevant to the bar plot (*p* < 0.05). White circles identify pairs of pipelines that differ on the presence/absence of the phase-based correction step. **(B,C)** Analysis of signal homogeneity within the cross-cortex lines. Homogeneity was calculated as the mean of the correlation and coherence between each pair of voxels [**(B)** shows the corresponding matrix for each pipeline, averaged through all samples], which was later averaged to report a single homogeneity value per pre-processing pipeline **(C)**. In the bar plot in **(C)**, the gray color helps to identify pipelines that included phase-based correction. The matrix above the bars identifies, in white, pairs of pipelines with significantly different homogeneity levels. White circles identify pairs of pipelines that differ on the presence/absence of the phase-based correction step. **(D)** Example of the regions subjected to cross-cortex line analysis for one volunteer. Different intensities in the heat color-map represent different cross-cortical lines, which extended from the CSF and up to the WM. In total, 280 lines were extracted from 13 subjects. Error bars represent the standard error of the mean.

Power spectra were computed by applying a Fourier transform to the data using Matlab. To evaluate frequency specific fluctuations of the signal power, spectra from the mean time course in the CSF, GM, and WM were calculated (Figure 2) and normalized by dividing each value of the spectrum by the maximum, for each subject. Spectral traces were then averaged among the thirteen subjects, and the result was normalized to a maximum of one for plotting. Power-spectrum plots are thus not intended to show the absolute power of different tissues, but rather compare the relative power of signals pre-processed with different pipelines at different frequencies. To facilitate comparison, a graph was created with the average power profile of the GM signal for all of the pre-processing pipelines. For comparison between the GM and CSF tissue, the relative power within the 0.015–0.04 Hz range (typical of neuronal fluctuations) with respect to the full spectrum was calculated in both compartments, rendering a measure of the fractional amplitude of very-low-frequency fluctuations (fAvLFF), and the GM/CSF ratio was computed. The average power spectrum covering frequencies between 0.01 and 0.1 Hz at each position within the cross-cortex line is shown in Figure 3A. The ratio of the power within the 0.015–0.04 Hz range (AvLFF) calculated in the GM and the CSF voxels and was used as an indicator of the level of signal localization in the GM.

To assess signal singularity/independence, the temporal correlation and the spectral coherence were averaged to give a measure of homogeneity for every pair of voxels along the cross-cortex line; this resulted in a 10 × 10 homogeneity matrix (Figure 3B). The level of homogeneity per pipeline was calculated as the average homogeneity across all voxels in the cross-cortex lines (Figure 3C), which constitutes an inverse measure of signal independence, i.e., lower homogeneity is equivalent to more independence or variability among the voxels.

Network probability maps were generated in FSL by performing independent component analysis using *melodic*. For visual inspection of the spatial specificity of the data, the component putatively assigned to the visual network, i.e., one of the strongest components, was selected. The mean functional image was used as the underlay, and the probability map was thresholded and presented as the overlay. The percentage of voxels surviving a 0.9 probability threshold was calculated and reported for each pre-processing pipeline.

To generate activation line profiles, a General Linear Model was applied to the evoked data to obtain beta and t-statistic activation maps using *3dDeconvolve* (AFNI). For group analysis, two regions of interest (ROI) were selected per task-fMRI scan, one covering the GM in the left pre-central gyrus, and the other covering the CSF adjacent to it. The degree of signal localization for each pipeline was assessed by thresholding the beta maps based on the t-statistic map (*p*-value = 0.001, *t*-value ≈ 4), averaging the beta values in the GM and CSF ROIs, and comparing the mean activation in the GM to that of the CSF. Paired *t*-tests were computed to obtain statistically significant differences across pipelines. To further assess the spatial localization of evoked responses in the cortical thickness, line profiles were computed from the activation maps related to a finger motor task and a finger motor + sensory task, which were acquired from one of the volunteers in the same fMRI session. Briefly, twenty lines, each consisting of thirty sampling points, were drawn over a portion of the left pre-central gyrus to detect the cortical activity evoked by movement/touch of the contralateral limb, and the average intensity profile of each activation map (beta map) along the lines was computed using a customized Matlab script.

## Results

As presented in Figure 1, the aim of the study was to evaluate signal specificity upon pre-processing of high-resolution GE-fMRI data with several existing methods. In the following, the results of an assessment at multiple levels are described.

### Contribution of Brain Tissue Types to the fMRI Spectrum Depending on Pre-processing

In order to characterize the specificity of the fMRI signal to the GM following the different pre-processing methods, the average signal from CSF, GM and WM—extracted from segmentation of the mean functional images—was subjected to spectral decomposition analysis. The graphs in Figure 2A show the average fMRI signal power distribution at different frequencies between 0.01 and 0.1 Hz, normalized to the maximum value of the spectrum for the 12 different pre-processing pipelines. The insets in the upper right corner of each sub-panel show the whole spectrum (from 0.001 to 0.14 Hz), and a gray rectangle has been positioned over the part of the spectrum that is magnified in the main graphs. Before pre-processing, the fMRI signal of all brain tissues was biased toward low frequencies, presumably due to hardware noise (pre-proc #1, see left peak in the minimized spectrum plot). This sub-0.01 Hz predominance was canceled by applying a band-pass filter (0.005–0.12 Hz), which shifted the maximum power to frequencies between 0.01 and 0.04 Hz, i.e., the typical range associated with resting-state activity (Zuo et al., 2010; Bajaj et al., 2014; Xue et al., 2014; Yuen et al., 2019). The capability of the pre-processing methods to strengthen the signal relevant to neuronal somata (0.015–0.04 Hz in GM) was assessed based on the distribution of the spectral power in the GM (Figure 2B) and by computing the mean power within this frequency range relative to the whole spectrum, i.e., the fractional amplitude of very-low-frequency fluctuations (fAvLFF) in the GM and in the CSF region, for comparison (Figures 2C,D). In the un-pre-processed data, the signals in the GM tissue exhibited a maximum power well below 0.01 Hz (only realigned, i.e., pipeline #1). With pipelines only involving temporal filtering (#2 and #6) the maximum power was ~0.01 Hz; when motion parameter regression was added or when CSF/WM regression was performed (#3, #7, #11, and #12) it was ~0.02 Hz; and in data subjected to physiological regression and partial volume regression, with or without phase-based correction (#4, #5, #8, #9, and #10) it was ~0.02–0.04 Hz. Of note, the power within the ~0.015–0.04 Hz range was considerably enhanced when phase-based correction was added to pipeline #2 (pipeline #6) (pale blue dashed line vs. solid line in Figure 2B). When comparing the relative power within the 0.015–0.04 Hz frequency range in GM and in CSF, it was observed that pipelines including physiological regression, especially following phase-based correction (pipeline #8) rendered the best results, with GM fAvLFF > CSF fAvLFF (Figures 2C,D). An exception was the realigned-only data (pipeline #1), which exhibited a much higher GM/CSF fAvLFF ratio, presumably due to the large CSF signal fluctuation at frequencies below 0.01 Hz in non-pre-processed data. Figure 2C shows that the phase-based correction step enhanced the activity in the GM relative to CSF when added to any of the implemented pipelines (dashed lines vs. solid lines); however, this increase was not statistically significant (Figure 2D).

The spectral analysis of fMRI data acquired during task performance is shown in Supplementary Figure 3. In comparison to the resting-state data, there was a stronger fMRI component fluctuating at ~0.05 Hz in evoked-fMRI scans, which corresponds to the frequency of the task paradigm (1/21 s).

### Signal Independence During Rest

To further investigate the potential benefits of the different pre-processing pipelines in laminar studies, for the scan of each participant, multiple ten-voxel lines were sampled crossing the cortical ribbon in areas of the frontal, parietal and occipital lobes (Figure 3D). A power-decomposition analysis was performed for each voxel within the cross-cortex line after pre-processing with the twelve different approaches (Figure 3A). CSF voxels exhibited the highest power across all frequencies (upper part of Figure 3A). However, this was less accentuated in the pre-processing pipelines that included physiological regression and partial volume corrections (#4, #5, #8, #9, and #10) (Dukart and Bertolino, 2014). The matrix on the right of Figure 3A shows the significant differences between pipelines in terms of ratio of AvLFF in the GM with respect to the CSF. Importantly, the phase-based venous correction method significantly improved the localization of 0.015–0.04 Hz signals within the GM (pairs of equivalent pipelines with and without phase-based correction are marked with a white circle in the significance matrix; note that all presented a *p*-value < 0.05). Smoothing (#10) resulted in a decreased power over all frequencies, presumably as a result of averaging among neighboring voxels.

The degree of signal dependence along the cross-cortex line was evaluated by measuring the correlation and coherence between pairs of voxels in the cross-cortex lines. These two metrics were averaged to generate one homogeneity matrix (Figure 3B), and the mean homogeneity across all pairs of voxels rendered a single homogeneity value per pre-processing approach (Figure 3C). The highest level of heterogeneity between the voxels across the cross-cortex line, indicating a better signal separation across the cortical thickness (lowest homogeneity), was obtained with pre-processing pipeline #9, where the phase-based correction method was applied to data after regression of motion parameters, pulse, breathing and average CSF and WM signals. The significant differences between pre-processing pipelines in terms of signal homogeneity are presented in a significance matrix in Figure 3C, where white cells represent significant differences between pairs. As expected, smoothing with 1 mm Gaussian filter kernel rendered the most homogeneous signals across the cortex, followed by the pipeline including only realignment. Phase-based correction significantly decreased the homogeneity levels in all pipelines, demonstrating the advantage of adding this step in high-resolution applications. Pipelines differing only in the presence or absence of phase-based regression are connected with a light gray line in the bar plot (Figure 3C) and shaded with a different intensity to facilitate comparison.

**Figure 4:**
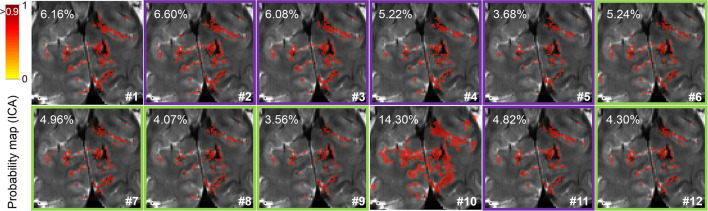
Resting-state network identification. Probability map of the visual network detected by independent component analysis focused on the visual cortex (cuneus) in a representative subject. The percentage of voxels surviving a 0.9 probability threshold (image overlay) is indicated in the upper left corner for each pre-processing pipeline. Green and purple frames surrounding some maps highlight pipelines with or without phase-based correction, respectively.

For visual inspection of the spatial localization, a resting-state network probability map of a portion of the visual cortex of one volunteer is presented in Figure 4. The improved signal specificity achieved with more complete pre-processing—excluding smoothing—can be clearly observed as a reduction in the percentage of voxels surviving a 90% probability threshold in pipelines #5 and #9, which corresponds to the finer delineation of the resting-state network. Here, it can also be observed that phase-based correction improves the spatial specificity of signals in all the assessed pipelines (compare the probability maps #2, #3, #4, #5, and #11 to their corresponding phase-based corrected versions, #6, #7, #8, #9, and #12, respectively).

### Evoked Activation

**Figure 5:**
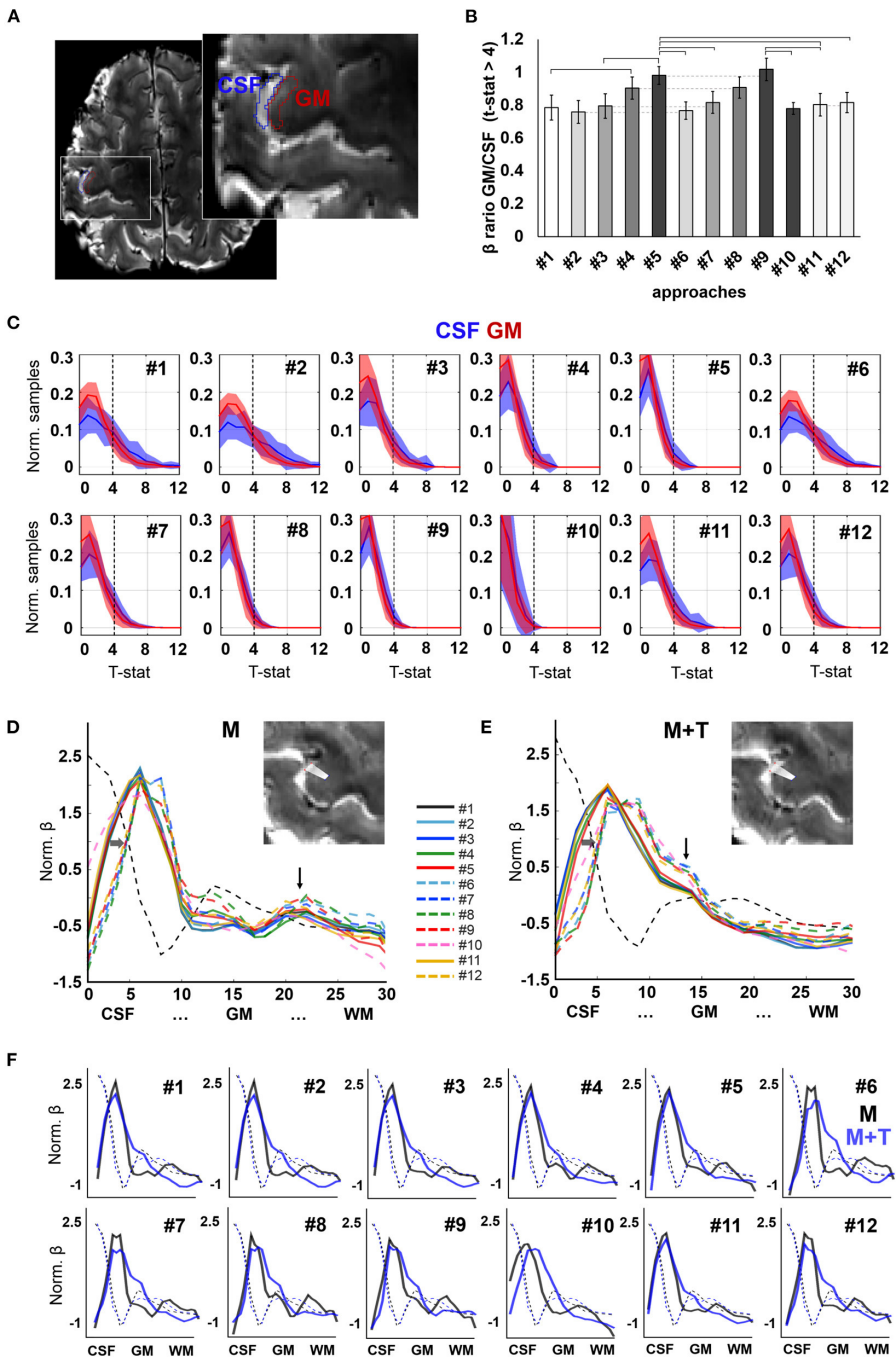
Evoked activation. **(A)** Axial slice showing the delineation of CSF and GM ROIs in the pre-central gyrus of a representative participant, used for group analysis. **(B)** Ratio of the mean beta-value across active voxels (t-statistic > 4) in GM with respect to CSF. Different intensities are employed to facilitate identification of pairs that differ only in the absence or presence of phase-based correction. Error bars represent standard error of the mean. Horizontal lines signal significantly different pairs, with *p*-value <0.05. *N* = 13 volunteers. **(C)** Histograms showing the distribution of t-statistic across voxels within the CSF and GM ROIs. Shaded areas represent the standard deviation of the mean across participants (*N* = 13). **(D,E)** Activation profiles observed in the left pre-central gyrus of a subject performing a motor task [“M”, **(D)**] and a motor task involving touch [“M+T”, **(E)**]. Vertical arrows point to a salient peak observed in deep layers during the motor-only task and intermediate layers in a motor + sensory task. Horizontal arrows highlight the shift of the first activation peak, near the CSF, toward the GM upon phase-based correction. **(F)** Activation profiles of both tasks (motor, in black, and motor + touch, in blue) overlaid on the same graph for each pre-processing pipeline. Background dashed lines indicate the intensity profile of the background image for each scan (averaged T2*-weighted), useful to assess alignment between the sampling lines used in both scans.

To assess the spatial specificity of evoked fMRI, here a motor task performed with the right index finger, the activation maps of the thirteen subjects were studied in two ROIs covering either a portion of the GM or the CSF in the left pre-central gyrus (M1 contralateral to the active limb) (Figure 5A). The beta maps were first thresholded (voxels with *p*-value < 0.001 were removed), and the surviving voxels were averaged within the GM or within the CSF ROI. The ratio of the GM and CSF beta-values is shown in Figure 5B and, with the exception of pipeline #1 and #10, in Table 1. Figure 5C shows the mean ± standard deviation of the distribution of the t-statistic in GM and CSF for each pre-processing pipeline across the thirteen volunteers. The fMRI data achieved higher significant beta values in the GM with respect to CSF when physiological signal regression was added to the pre-processing pipeline (this increase was significant compared to most other ROIs) (Figure 5B). Although not statistically significant, an increased GM/CSF ratio could be observed following the addition of phase-based correction to most pre-processing pipelines. Table 1 shows the activation ratio obtained from pipelines #2, #3, #4, #5, and #11, i.e., before phase-based correction, and in #6, #7, #8, #9, and #12, i.e., after phase-based correction, and provide the percentage ratio between both sets of pipelines.

**Table 1:** GM/CSF activation ratio in terms of mean beta coefficient across significant voxels.

**Previous** **pre-processing**	**Beta GM/beta CSF** **before phase-correction**	**Beta GM/beta CSF** **after phase-correction**	**% change**
+ filtering (#2–6)	0.76 ± 0.25	0.77 ± 0.19	+ 0.96%
+ motion r (#3–7)	0.80 ± 0.27	0.82 ± 0.24	+ 2.77%
+ physio r (#4–8)	0.90 ± 0.24	0.91 ± 0.24	+ 0.38%
+ CSF/WM r (#5–9)	0.98 ± 0.19	1.02 ± 0.25	+ 3.82%
(#11–12)	0.80 ± 0.25	0.82 ± 0.22	+ 1.59%

In order to assess the influence of pre-processing on the delineation of evoked cortical activation profiles, data obtained during two neurophysiologically-different tasks were analyzed in the same cortical segment in one volunteer who first performed movement of one finger, i.e., a motor task (“M”), and, later, movement that involved touching (“M + T”), i.e., adding somatosensory information processing. Upon movement, analysis of the evoked activity in the pre-central gyrus demonstrated a profile with strong activation in the cortical surface and a second peak occupying deep layers of the cortex, consistent with the involvement of motor efferents (Figure 5D). In contrast, the addition of sensory processing attenuated the activation of the deep layers and enhanced the signal in the intermediate layers of the cortex (Figure 5E). The activation profiles of both tasks are shown overlaid in the graphs shown in Figure 5F. Although the activation peaks corresponding to somato-motion and somato-sensation were observed in most pipelines, a better identification was achieved by adding phase-based correction, which also shifted the mean activation peak away from the CSF territory (dashed lines vs. solid lines in Figures 5D,E).

## Discussion and Conclusions

Given the recent interest in investigating laminar functional dynamics, new fMRI sequences have been developed that exploit diverse contrasts to study brain function with high spatial resolution (Feinberg et al., 2018; Huber et al., 2018, 2020a; Kashyap et al., 2018b; Berman et al., 2020; Chai et al., 2020; Yun et al., 2020). Of those, sequences based on GE BOLD entail notable advantages due to their lower specific absorption rate, higher signal-to-noise ratio and faster acquisitions (Gati et al., 1997; Fukuda et al., 2021; Weldon and Olman, 2021), resulting in robust functional images with unprecedented coverage and resolution (e.g., Yun et al., 2020). However, these sequences are generally more biased to field inhomogeneities, such as those emerging from the venous vasculature; hence, pre-processing to substantially diminish signal contamination constitutes a critical step if the resulting images are to be analyzed in a laminar context. In this work, the performance of high-resolution GE BOLD fMRI pre-processed with twelve different pipelines was evaluated, principally in terms of signal localization, both in resting-state and task conditions. We did not provide a novel method to pre-process fMRI, instead, we offered a series of evaluations to understand how the existing approaches benefit or not GE-data for high-resolution applications. Pre-processing pipelines included elemental steps that are part of every fMRI analysis as well as more specific methods that are especially beneficial for high-resolution data, which had an important impact on signal localization, based on our results. Global signal regression was not performed in any of the pipelines because the meaning of what constitutes an average brain signal remains an open discussion in the field and has been the focus of multiple publications (Falahpour et al., 2013; Amemiya et al., 2016; Mayhew et al., 2016; Thompson et al., 2016; Hung and Liu, 2017; Liu et al., 2017; Murphy and Fox, 2017; Belloy et al., 2018; Spreng et al., 2019; Aquino et al., 2020; Umeh et al., 2020). As expected, the noise related to known sources (e.g., head motion, respiration or heart beating) was substantially reduced by applying routine fMRI pre-processing steps (the results can be found in Supplementary Figure 2; Supplementary Note 1). Similar to the reduction of noise and to some extent also expected, the signal localization on the GM with respect to CSF, as well as the signal independence along the cortical depth was improved with more complete pre-processing. Importantly, the addition of phase-based correction produced a consistent enhancement of signal localization in every measure assessed (e.g., yielding a better localization of resting-state and evoked data in GM and decreasing homogeneity among neighboring voxels). This indicates an advantage with respect to non-phase-corrected approaches which should be taken into consideration in high-resolution studies based on GE-fMRI. The efficiency of the phase-regression step was, to a certain extent, anticipated, as the phase signal is especially sensitive to magnetic field inhomogeneities derived from any source of motion and from the deoxyhemoglobin in large venous vessels (Lai et al., 1996; Menon, 2002, 2012). The phase-based correction method applied in some of the pre-processing pipelines subtracts the amount of magnitude signal that can be explained by its corresponding phase from the magnitude data. After pre-processing, voxels covering the vasculature should reflect the activity changes of neighboring neurons. While changes in both magnitude and phase images in large vessels can be detected upon neuronal activation, the magnitude signals predominate in the microvasculature, and hence, removal of the phase contributions after pre-processing can help optimize signal localization, specifically in the gray matter, where the microvasculature prevails. By removing the phase-related signals, a partial reduction of the contamination that emerges from motion and respiration has also been previously reported (Barry et al., 2010). Other methods have been proposed to eliminate the contribution of veins to the data; one example is the automatic exclusion of venous voxels (Barth and Norris, 2007; Koopmans et al., 2010). Although this segmentation eliminates the surface-vein bias in cortical areas where vessels can be detected (e.g., as intravoxel dephasing in sequences sensitive to veins or as low intensity in common T2/T2^*^ images), voxels covering veins that are not easily identified (e.g., if using only T2/T2^*^ contrast) will remain in the analysis. Importantly, even assuming complete eradication of venous voxels, the blooming effect, i.e., the field inhomogeneities that extend to neighbor voxels, will persist in the intact gray matter. Additionally, the presence of ascending veins, collecting the blood from venules in deeper territories toward downstream vessels, would still lead to a predominance of BOLD signals near the surface. Simulations with 0.75 mm voxels have estimated that up to 40% of the cortical depth (measured from the surface) is contaminated by the effect of pial veins (Kashyap et al., 2018b), and the point-spread function of BOLD signals measured along the cortical depth was estimated as 20% of the cortical thickness (Havlicek and Uludag, 2020). For a review on the dependence of the BOLD signal on the vasculature and the associated effects in laminar fMRI, see references (Uludag and Blinder, 2018; Fukuda et al., 2021). Due to the complexity of BOLD, both in terms of its nature and the vascular arrangement in the cortex, voxel-wise correction methods offer a good alternative to clean up the fMRI signal when the vascularity of the cerebral cortex cannot be sufficiently mapped (e.g., in human whole-brain laminar applications). Besides the phase-based correction method introduced by Menon (2002), spurious signals influenced by cerebral veins can be detected as delayed responses of higher phase amplitude and removed at a voxel level from the fMRI data (Lee et al., 1995; Kay et al., 2020). However, this method is not applicable to resting-state paradigms as these are not subjected to predicted responses. Additionally, several models have been developed that can be used to de-noise task-fMRI data from vascular signals (Heinzle et al., 2016; Markuerkiaga et al., 2016; Fracasso et al., 2018; Kashyap et al., 2018a), but these cannot be applied to, for instance, connectivity analysis or resting-state data, due to the same reason as above (not subjected to predicted responses). Importantly, the organization of the intra-cortical vasculature is not homogeneous, i.e., it does not follow the alignment of the neurons at the different cortical layers but rather responds to metabolic demands (Borowsky and Collins, 1989; Weber et al., 2008; Blinder et al., 2013), thus introducing additional bias to general models. Hence, the phase-correction method constitutes a good alternative to correct high-resolution resting-state data in a model-free voxel-wise manner.

The effect of smoothing at high field, both directly and as a consequence of steps like motion correction, has been previously investigated in the context of functional activation of small deep brain nuclei (Murphy et al., 2020). Although smoothing can help to reduce noise while preserving true BOLD signals, it has a detrimental effect on the signal spatial specificity, e.g., it increases the partial volume effect. Hence, applications like laminar fMRI, where the voxel size is typically bigger than the desired target resolution, do not benefit from common smoothing procedures. However, smoothing restricted to individual cortical surfaces and other advanced smoothing methods are promising strategies to enhance signal cleaning while maintaining a level of specificity within the cortical ribbon that is convenient for the given application (Hagler et al., 2006; Blazejewska et al., 2019; Brodoehl et al., 2020).

Co-registration of functional images to an anatomical scan introduces a certain blurring into the functional images, since the anatomical scan is typically acquired with a different imaging sequence (e.g., MPRAGE/MP2RAGE). For this reason, laminar fMRI tends to either project the anatomical image to the functional data or substitute the MPRAGE-based anatomical scan with the functional sequence-based scan reconfigured to yield T1 contrast (i.e., with good GM/CSF/WM contrast) (Kashyap et al., 2018a). The results shown here were all collected from functional data only, i.e., no co-registration was applied to the data and tissue segmentation was directly performed on the mean functional image computed after realignment.

The acquisition of line activation profiles evoked by two different tasks in one volunteer served to demonstrate the ability of the GE-sequence to differentiate somato-sensation from somato-motion based on depth-dependent responses. Here, the most complex pipelines with added phase-regression clearly identified the contribution of the deep layers of the motor cortex to the processing of the motor task. This presumably represents inputs from premotor and supplementary motor areas and coincides with the efferent motor fibers toward ponto-medullary nuclei and motor thalamus. When the task included sensory processing, additional involvement of the intermediate layers was detected, possibly indicating cross-talk between primary motor and primary sensory areas of the cortex and reflecting the modulation by afferent sensory thalamic fibers that alter motor behavior (Weiler et al., 2008; Mao et al., 2011; Hooks et al., 2013; Huber et al., 2017). In both tasks, activation of the superficial layers may respond to inputs from the motor thalamic nuclei, although a spurious contribution from the vasculature is likely to have remained despite high-level pre-processing. Interestingly, phase-based correction shifted this superficial peak toward deeper locations, indicating an effective de-veining effect.

The dataset employed here to evaluate the effect of pre-processing in high-resolution applications used voxels of size 0.63 × 0.63 × 0.63 mm, i.e., 0.25 mm^3^. This is in line with the voxel size employed by the community in evoked laminar fMRI and well below the resolution achieved by most whole-brain fMRI studies; hence, our results could be transposed to most GE sequences intended to perform laminar assessment of brain function. However, it is worth noting that the sequence employed here (EPIK) is characterized by a robust behavior against distortions (Yun et al., 2013) and data obtained with common EPI are expected to have larger geometric distortions caused by susceptibility differences. The temporal resolution of the presented data (TR = 3.5 s) is in the range to investigate resting-state activity or responses corresponding to block-paradigms. Data acquired at higher rates could benefit from further pre-processing, i.e., by using statistical models that allow detection and regression of sources of physiological contamination (Agrawal et al., 2020). Other methods that have proven valuable to reduce fMRI noise in the literature [e.g., more advanced motion-correction procedures (Power et al., 2018)] are expected to further benefit high-resolution data, as signal specificity is usually enhanced upon reduction of fMRI noise. Our analysis demonstrates that GE data acquired with sub-millimeter spatial resolution is particularly sensitive to the applied pre-processing and that smoothing-free routine fMRI cleaning methods with combined phase regression (to reduce vein bias) significantly improve the definition of depth-dependent activation patterns.

## Data Availability Statement

Access to the raw data supporting the conclusions of this article can be made available by the corresponding author(s) upon reasonable request, without undue reservation.

## Ethics Statement

The studies involving human participants were reviewed and approved by the Local Institutional Review Board (RWTH Aachen University, Germany). The participants provided their written informed consent to participate in this study.

## Author Contributions

NJS invented the original EPIK sequence. SY developed the MR imaging sequence and the corresponding image reconstruction software. SY and PP-R performed *in vivo* experiments. PP-R designed and performed data analysis, wrote the manuscript, and prepared figures. SY and NJS reviewed the manuscript. All authors contributed to the article and approved the submitted version.

## Funding

This study was funded by internal, institutional resources (Institute of Neuroscience and Medicine 4, Forschungszentrum Juelich). Open access publication was partially funded by the Helmholz Association.

## Conflict of Interest

The authors declare that the research was conducted in the absence of any commercial or financial relationships that could be construed as a potential conflict of interest.

## Publisher's Note

All claims expressed in this article are solely those of the authors and do not necessarily represent those of their affiliated organizations, or those of the publisher, the editors and the reviewers. Any product that may be evaluated in this article, or claim that may be made by its manufacturer, is not guaranteed or endorsed by the publisher.
