# Supplementary material for: Pre-processing of Sub-millimeter GE-BOLD fMRI Data for Laminar Applications
Source: Front Neuroimaging. 2022 May 4;1:869454. doi: 10.3389/fnimg.2022.869454 (PMC10406219; doi:10.3389/fnimg.2022.869454)
Supplement: Supplementary file 1 [file Data_Sheet_1.PDF]

Supplementary material for the manuscript:

Pre-processing of sub-millimetre GE-BOLD fMRI data for laminar applications

Authors

Patricia Pais-Roldán[1], Seong Dae Yun[1], N. Jon Shah[1-4 *]

[1]Institute of Neuroscience and Medicine 4, Medical Imaging Physics, Forschungszentrum Jülich, Germany

[2]Institute of Neuroscience and Medicine 11, Molecular Neuroscience and Neuroimaging, JARA, Forschungszentrum Jülich, Germany

[3]JARA - BRAIN - Translational Medicine, Aachen, Germany

[4]Department of Neurology, RWTH Aachen University, Aachen, Germany

Running Title: Pre-processing for GE-EPIK laminar fMRI

*Corresponding Author:
Prof. N. Jon Shah
Institute of Neuroscience and Medicine-4,
Research Centre Jülich,
52425 Jülich,
Germany
Tel.: +49 2461 61 6836
Email: n.j.shah@fz-juelich.de

This document contains:

Supplementary Note 1

Figure S1

Figure S2

Figure S3

Supplementary Note 1:

Contribution of non-neuronal sources to the fMRI signal

In order to assess the contribution of noise to high-resolution GE fMRI signals, a correlation analysis was first performed on forty-seven regressors of no interest and the magnitude and phase fMRI time courses of ten voxels crossing the cerebral cortex (**Fig. S1**). Potential regressors of no interest included signals related to motion, physiological signals, and the mean time course of the voxels within the grey matter (GM), white matter (WM) and cerebrospinal fluid (CSF). Based on ten individual voxels crossing the cortical ribbon at multiple locations and from the average GM, WM and CSF brain regions, a strong temporal correlation was observed between the motion parameters and the time course of the magnitude and phase of the fMRI signal.

Next, we analysed the level of signal contamination due to motion, physiological interference and noise present in the global CSF, GM or WM tissue signal in 12 versions of the fMRI data, each pre-processed with a particular pipeline. The series of pre-processing methods involved the typical rs-fMRI pre-processing steps, i.e., realignment (#1), a combination of realignment with added temporal filtering (#2), with motion parameter regression (#3), with physiological signal regression (#4) and with regression of the mean CSF and WM signal (#5). Additionally, a step to correct for GE-based inhomogeneities (typically related to veins, i.e., relevant in cortical-depth analysis) was added, which consisted in removing any residual signal variance that could be explained by the phase component of the MRI signal (pipelines #6-#9). To assess the effects of smoothing, a 1mm Gaussian blur was applied to one of the pipelines (#10). To distinguish the effect of physiological regression from that of the partial volume correction, we incorporated a pre-processing pipeline with regression of CSF and WM signals but without physiological regression, with and without the phase-based correction step (#11 and #12). The twelve pre-processing approaches are presented in **Figure S2A**. The degree of contamination in each set of pre-processed fMRI data caused by the different noise sources is shown in **Figure S2B**. As expected, the highest level of contamination was detected in fMRI data that had only been subjected to realignment (#1). These data exhibited large amounts of signal correlated with motion and partial volume effects. Both the partial volume effects and the contamination from head motion were substantially reduced with additional band-pass filtering, suggesting that a large portion of the noise in the fMRI signal is out of the typical range of the hemodynamic response function (HRF) spectrum relevant to neuronal activity. The presence of noise related to pulse and respiration (“physio”) was clearly reduced when incorporating physiological regression by means of RETROICOR (pre-proc #4, #5, #8, #9, #10). No apparent effect was observed in terms of the average signal correlation with motion, physiology or CSF/WM when the phase-based correction method was applied to the data (i.e., #6, #7, #8, #9, #12 vs. #2, #3, #4, #5, #11, respectively). Overall, the pre-processing pipeline combining motion-parameter regression, physiological regression and regression of CSF and WM (pre-proc #5 and its variations #9 and #10) was the most effective in reducing noise from the fMRI signal. The correlation results suggest that all the standard pre-processing methods proposed in common fMRI studies should be applied to high-resolution data to minimize the influence of known sources of noise.

Figure S1.

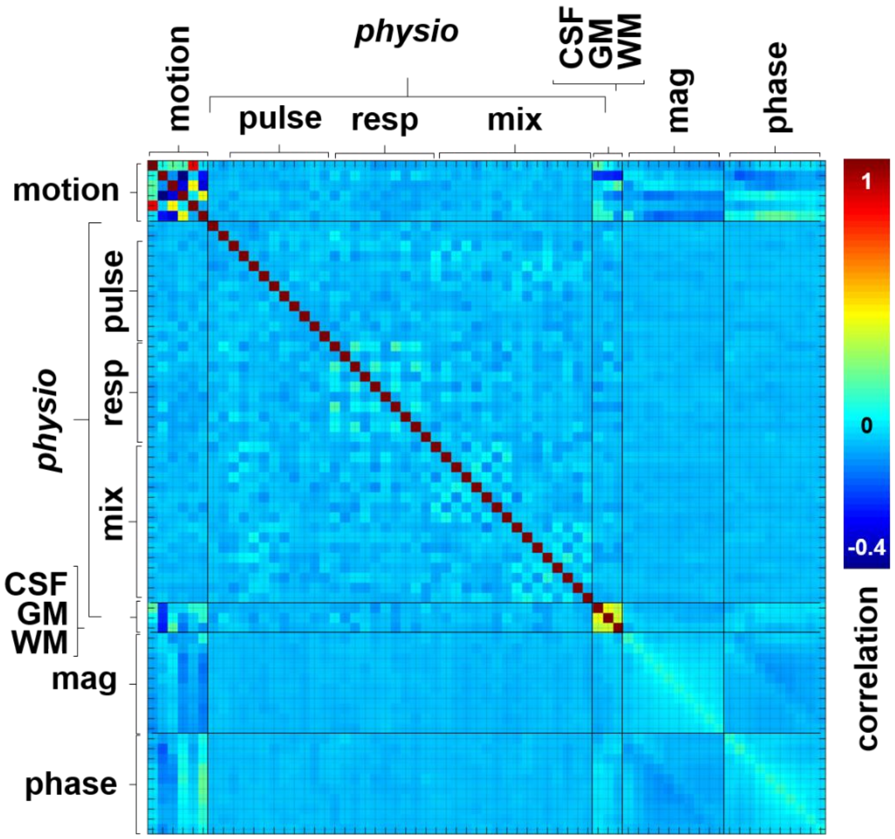

Figure S1. Potential sources of noise in the fMRI data and their relationship. Matrix showing the mean temporal correlation between potential sources of noise in the data and the mean magnitude and phase time courses at ten voxels crossing the cerebral cortex from CSF to WM (“mag” and “phase” respectively).

Figure S2.

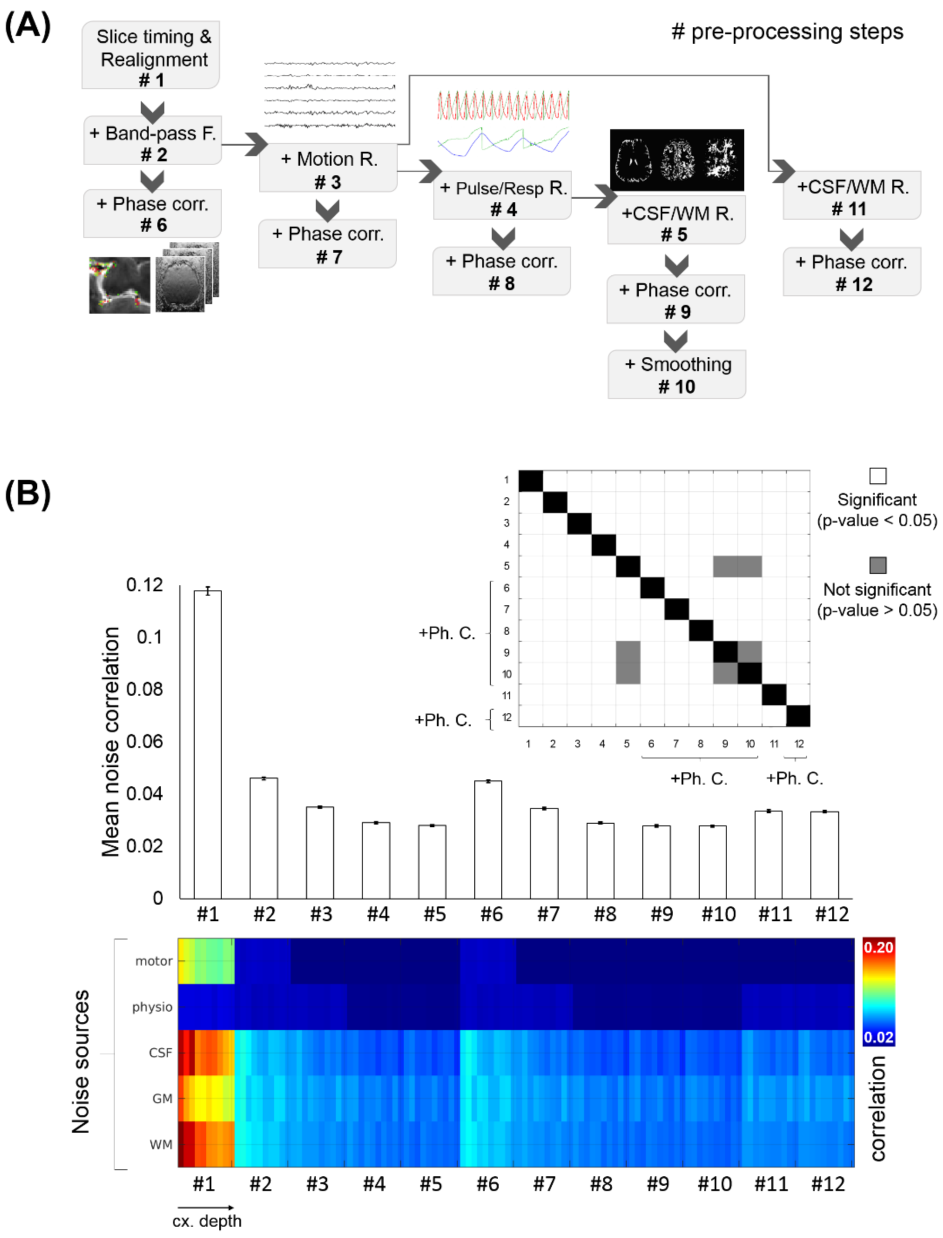

Figure S2. Pre-processing approaches and the influence of noise on the fMRI data. (A) Pre-processing pipelines. Pre-proc. #N, with N=1..5 refers to a pipeline that includes all the previous steps up to N. Pre-proc. #6 to #9 add phase-based vein correction to the previous pipelines. Pre-proc. #10 adds smoothing to the corrected data #9 for comparison. Pre-proc #11 and #12 add partial volume correction and phase-based correction, respectively, to Pre-proc #3. **(B) Top:** bar plot showing the mean correlation between noise sources and the voxel fMRI signal pre-processed with the different pipelines. The matrix above identifies, in white, pairs of pipelines with significantly different levels of correlation with noise. Pipelines including phase-regression correction are labelled with "+Ph. C.". **Bottom:** Matrix showing the temporal correlation between the potential sources of noise and the magnitude signal in ten voxels crossing the cortical ribbon after correction with twelve different pre-processing approaches. Letters indicate significantly different groups ($p<0.005$ in all cases). Error bars indicate standard error of the mean. N=13 healthy volunteers.

Figure S3.

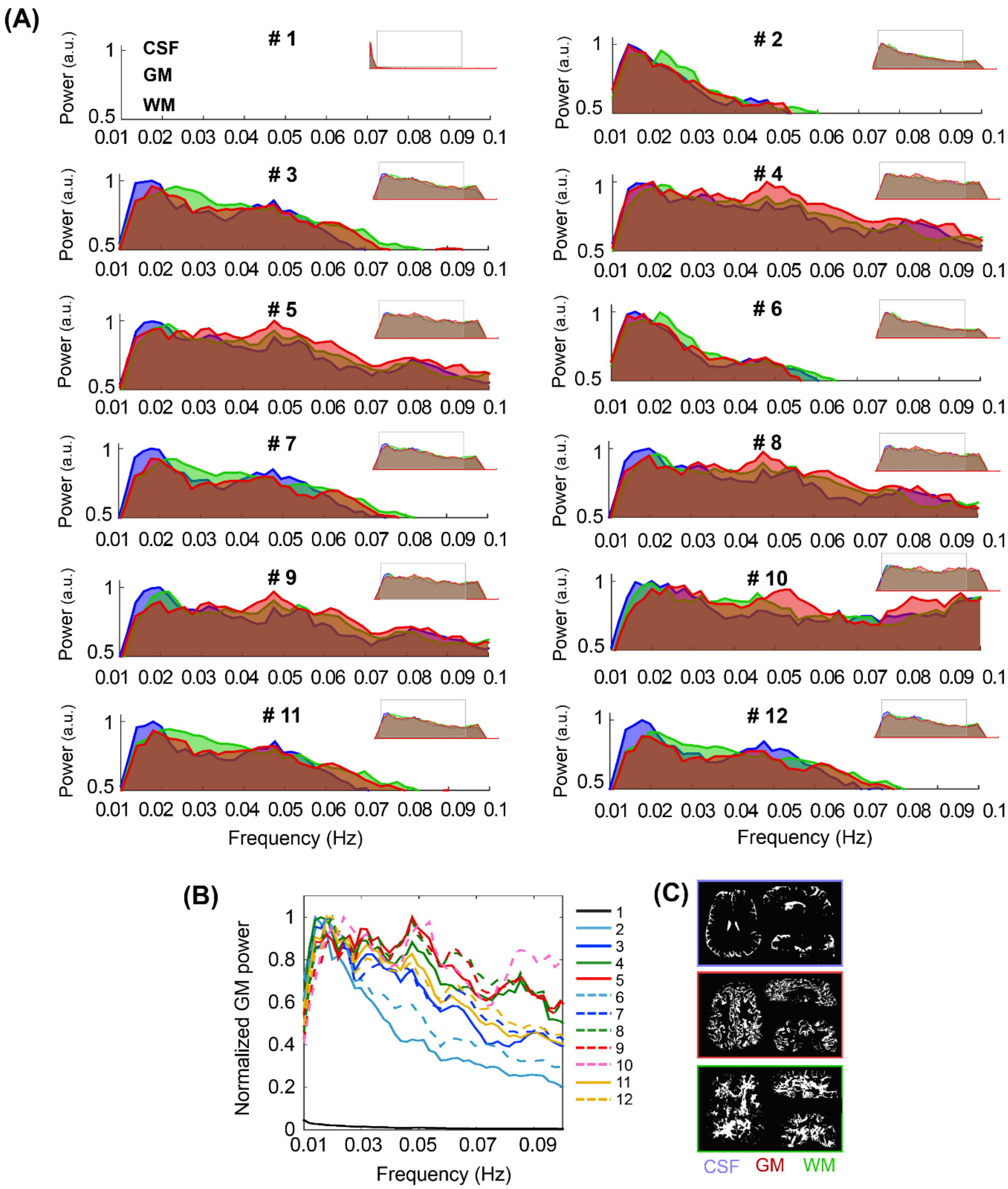

Figure S3. Spectral characteristics of CSF, GM and WM following different pre-processing methods. (A) The graphs show the frequency-power decomposition of the average CSF, GM and WM time course resulting from applying different pre-processing steps to a task fMRI scan in 13 participants. **(B)** The graph shows the normalised power of the average GM time course pre-processed with different approaches. Note that the normalised signal in Pre-proc. #1 (black line) is near zero due to having its maximum below 0.01Hz. **(C)** Example of the tissue-specific masks generated automatically from the mean functional image in one volunteer. N=13 healthy volunteers.
